# Ten-step minimally invasive slalom unilateral laminotomy for bilateral decompression (sULBD) with navigation

**DOI:** 10.1186/s12891-023-06940-7

**Published:** 2023-11-02

**Authors:** Siegfried J. Adelhoefer, Jessica Berger, Catherine Mykolajtchuk, Jaskeerat Gujral, Blake I. Boadi, Brian Fiani, Roger Härtl

**Affiliations:** Department of Neurological Surgery, Weill Cornell Brain and Spine Center, Weill Cornell Medicine and New York Presbyterian Hospital - Och Spine, 525 E 68th St, Box 99, New York, NY 10065 USA

**Keywords:** Minimally invasive spinal surgery, Unilateral laminotomy for bilateral decompression, Laminotomy, Tubular decompression, Lumbar spinal stenosis, Navigation, Microdiscectomy

## Abstract

**Background:**

Unilateral laminotomy for bilateral decompression (ULBD) is a MIS surgical technique that offers safe and effective decompression of lumbar spinal stenosis (LSS) with a long-term resolution of symptoms. Advantages over conventional open laminectomy include reduced expected blood loss, muscle damage, mechanical instability, and less postoperative pain. The slalom technique combined with navigation is used in multi-segmental LSS to improve the workflow and effectiveness of the procedure.

**Methods:**

We outline ten technical steps to achieve a slalom unilateral laminotomy for bilateral decompression (sULBD) with navigation. In a retrospective case series, we included patients with multi-segmental LSS operated in our institution using the sULBD between 2020 and 2022. The primary outcome was a reduction in pain measured by Visual Analogue Scale (VAS) for back pain and leg pain and Oswestry Disability Index (ODI).

**Results:**

In our case series (*N* = 7), all patients reported resolution of initial symptoms on an average follow-up of 20.71 ± 9 months. The average operative time and length of hospital stay were 196.14 min and 1.67 days, respectively. On average, VAS (back pain) was 4.71 pre-operatively and 1.50 on long-term follow-up of an average of 19.05 months. VAS (leg pain) decreased from 4.33 to 1.21. ODI was reported as 33% pre-operatively and 12% on long-term follow-up.

**Conclusion:**

The sULBD with navigation is a safe and effective MIS surgical procedure and achieves the resolution of symptoms in patients presenting with multi-segmental LSS. Herein, we demonstrate the ten key steps required to perform the sULBD technique. Compared to the standard sULBD technique, the incorporation of navigation provides anatomic localization without exposure to radiation to staff for a higher safety profile along with a fast and efficient workflow.

Lumbar spinal stenosis (LSS) must be suspected in patients over 50 years of age complaining of leg pain or paresthesia on walking. Conservative management is recommended as first-line in lumbar spinal stenosis. However, deteriorating weakness necessitates surgical intervention [1]. Minimally invasive spinal surgery (MISS) has been widely adopted as a compromise between reaching an effective solution to the patient’s symptomatology at the lowest invasiveness possible. The shift towards MISS is accelerated by patient preferences to be treated less invasively and by insurers emphasizing the need to provide quality care cost-effectively, and when medically possible in an ambulatory setting [2]. MISS has been shown to reduce surgical site infections as compared with open approaches [3, 4].

The open unilateral laminotomy for bilateral decompression was first described in 1997 as a surgical approach to achieve bilateral decompression via a unilateral entry point while preserving the spinous processes, the interspinous ligaments, and the physiological stability by the muscular apparatus. It was adapted for minimally invasive approaches in 2002 by Sylvain Palmer which now is what is referred to as unilateral laminotomy for bilateral decompression (ULBD) [5, 6]. The ULBD approach has previously been shown to reduce postoperative hospital recovery time, postoperative pain, and opioid use [7]. Intraoperatively, less surgical trauma is commonly caused when using the ULBD approach [8]. The ULBD approach is the preferred technique to treat symptomatic LSS as it achieves a long-lasting decompression [9]. Evidence is emerging that the ULBD is even superior in treating multilevel spinal canal stenosis [10].

Boukebir et al. have previously outlined the fundamental principles of MIS decompression: (i) the ULBD involves a unilateral entry point, “over-the-top” approach, and bilateral decompression, (ii) MIS surgery reduces the instability compared with open surgical approaches as damage to the muscular apparatus is reduced, and (iii) the slalom ULBD is the technique of choice for multi-segmental spinal stenosis using alternating entry points to further reduce the invasiveness of the decompression procedure [11]. Our group has also demonstrated the safety of using two microscopes during sULBD in multi-segmental LSS. Our rationale was to decrease the operative time and thereby the infection risk and the general anesthesia time [12].

The AO Foundation has outlined the ten surgical steps in microsurgical tubular discectomy and laminotomy. As in spinal surgery in general, the most common complication occurring is dural tears. In minimally invasive tubular surgery, dural tears may occur when the yellow ligament is removed [13, 14]. A curriculum roadmap in MISS has also been outlined previously: Briefly, standardized technique protocols and outcome metrics are required to deliver effective training and education in MISS [15]. The critical steps in unilateral laminotomy for bilateral decompression using a tubular approach have been agreed upon by a panel of 29 surgical experts. The metric-based procedure may shorten the learning curve associated with minimally invasive spinal surgery [16]. The metric-based surgical steps have also been used in a training setting: After developing the ten steps for MISS, we tested the utility of this protocol on RealSpine models by Realists Training Technologies (Leipzig, Germany) [17].

The ULBD was adapted to a “slalom” technique to target multisegmental LSS and was first described in 2013 [18]. The slalom ULBD (sULBD) is muscle-sparing and facet-joint-sparing and facilitates a faster postoperative recovery time, minimizes estimated blood loss, and shortens the length of stay (LOS). The “slalom” technique with multiple incisions is recommended in multisegmental lumbar spinal stenosis in order to reduce postoperative instability. In the “slalom” technique, laminectomies are approached from alternating sides to spare muscles and fascia for faster postoperative recovery. Risk of iatrogenic injury can be further minimised by passing the dilator through the subsequent laminectomy site from the opposite side. Postoperative improvements following the sULBD measured by Oswestry Disability Index (ODI), visual analogue scale (VAS) for back and leg pain have been shown previously [19].

Biomechanically, the advantage of ULBD is that it leads to less disruption of the posterior elements and therefore helps avoid iatrogenic instability that may happen after conventional open laminectomy. Open conventional laminectomy may require stabilization or fusion surgery in patients with LSS and spondylolisthesis who originally did not have frank instability [20].

Using “total navigation” we postulated it would improve the overall workflow, increase accuracy, and minimize radiation exposure to the surgical team as compared with fluoroscopy techniques, thereby also eliminates wearing lead aprons [21, 22]. Here, we sought to give a detailed description of the step-by-step technique of the sULBD with navigation using tubular retractors for the decompression of multisegmental LSS. We hypothesize that the invasiveness of the surgery can be further reduced in treating patients with multisegmental LSS by alternating incisions to leave the muscular apparatus intact as much as possible.

## Methods

### Study design and inclusion criteria

This is a single-center, retrospective study. Using current procedural terminology code for decompressive laminotomy from 2020 to 2022, patients were selected from our electronic charts. Patients were further screened for multisegmental LSS who subsequently underwent a sULBD procedure based on operative reports. We followed the three surgical principles of MISS: contralateral decompression, minimize instability, and indirect decompression. Inclusion criteria were 18 years or older, having 2 or more levels of decompression using the sULBD with navigation. Patients who required a fusion were not considered for this study. Thus, we excluded patients with multiple or combined disc herniations who required one or multiple fusions. The minimum follow-up period for this study was 9 months.

### Ethics approval and consent to participate

This study was conducted in accordance with local, national, and international guidelines and regulations. IRB approval was sought from the Weill Cornell Medicine Institutional Review Board for this study (IRB Protocol #: 19-120211199). Patients were carefully evaluated for eligibility for minimally-invasive spinal surgery. After a thorough clinical examination, informed consent to the planned procedure was obtained from all patients or their legal guardian(s). We have removed all potential identifiers (names, date of birth, etc.) from any imaging presented as part of this study. Informed consent was obtained from all subjects and/or their legal guardian(s) for publication of identifying information/images in an online open-access publication.

### Surgical technique

**Fig. 1:**
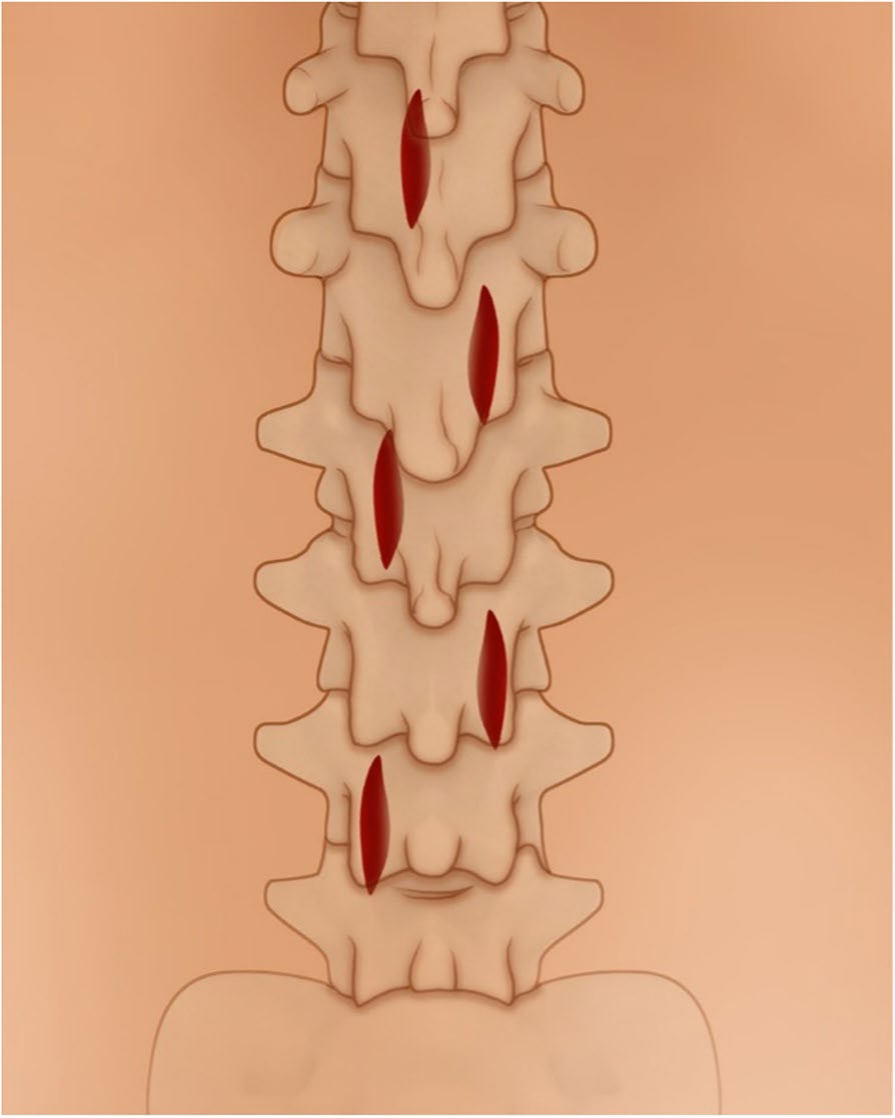
Alternating incisions on the posterior lumbar skin for the sULBD approach

**Fig. 2:**
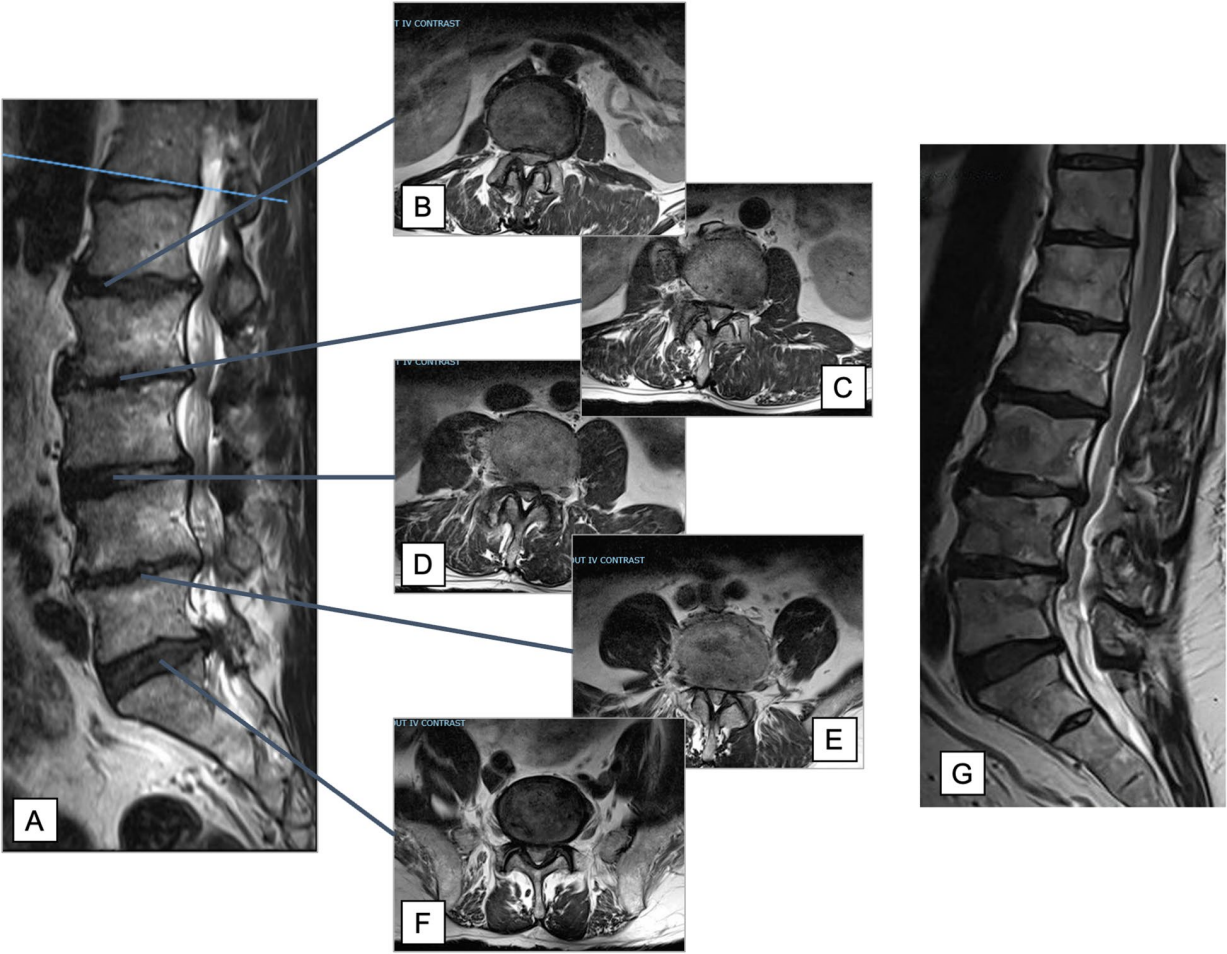
Case example of multilevel segmental lumbar spinal stenosis. Note the stenosis is located at the disc spaces and is non-continuous. **A** Sagittal view and (**B**-**F**) axial views of corresponding spinal levels. **G** Comparative MRI with continuous stenosis which would be better suited for open laminectomy than the sULBD. Abbreviations: MRI = Magnetic Resonance Imaging, ULBD = Unilateral laminotomy for bilateral decompression

The sULBD is a MIS surgical technique that is currently performed and taught at our institution. We developed an instructional ten-step guide for an “over-the-top” bilateral decompression via unilateral entry points on alternating sides (= giving rise to the association “slalom”) (Fig. 1). Initially, the patient is positioned and a low-dose CT scan is performed using Brainlab AIRO navigation with the reference array placed into the iliac crest opposite the side of the most caudal skin incision. One spin is performed per procedure and allows navigation for all levels. A control spin can be obtained after decompression to confirm correct levels and decompression. After a 3D spin has been obtained the skin incisions are planned using the navigated pointer and 3D navigation. An image viewer utilizing simultaneous imaging of saggital and axial cuts should be used intraoperatively (Fig. 2). Facet anatomy, the fluid signal of the joints, and bone spurs should be carefully assessed before tubular placing.

Typically one incision is made per level on alternating sides (= slalom). Depending on the individual anatomy and need for decompression sometimes 2 levels are treated via one incision. Using a 20-mm skin incision can give access to two segments – thus, separate fascial incisions are needed and the multifidus in between is left intact. It is important to dilate each level separately in order not to violate the multifidus muscle that inserts along the spinous process between adjacent levels. Skin incisions are planned approximately 1–2 cm lateral off midline at L4/5/S1 and more medially at higher levels to avoid violating the pars interarticularis. Following the incision, the tubular retractor is placed using sequential tubular dilators. The placing of the tubular retractor system is preceded by inserting subsequently larger diameters of dilators. The tube size used was 18 mm. However, a smaller tube size of 15 mm may be used to protect the pars interarticularis at higher levels (L1/2, 2/3, 3/4). This whole process is done using tactile feedback and the navigated pointer for orientation.

**Fig. 3:**
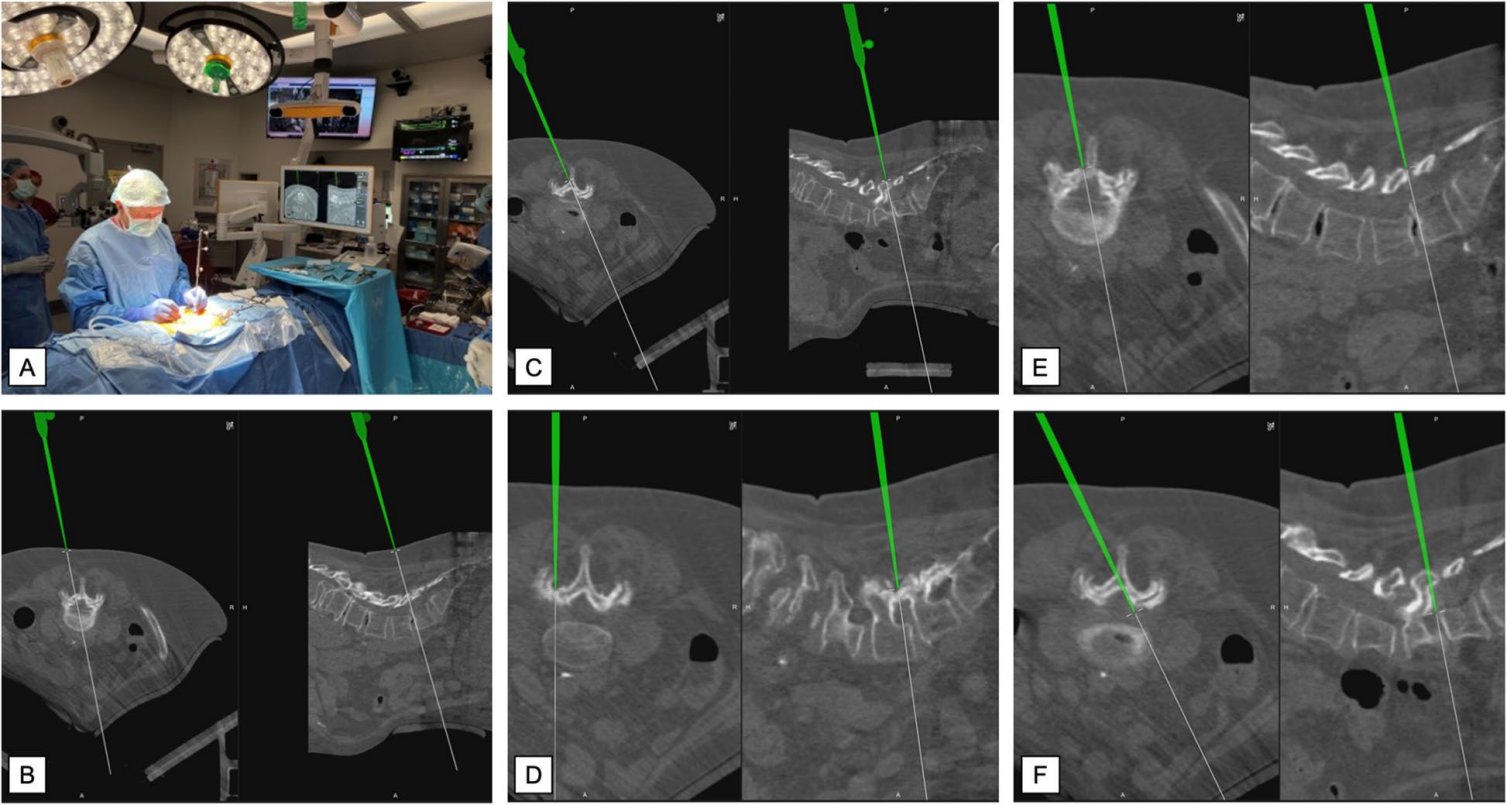
Navigation during the sULBD approach. **A** Planning incision for left L4-L5 ULBD using a sterile navigation pointer; **B** Skin incision using navigation, pointer placed on the skin before incision to confirm spinal level; **C** After placement of ipsilateral tubular retraction, confirmation of ipsilateral lamina using navigation is performed; **D** Navigation-confirmed pars interarticularis to minimize risk of injury to pars; **E** Drill placed at the inferior edge of the lamina with tip placed on ligamentum flavum; **F** Confirmation of the contralateral pedicle using navigation

Soft tissue is removed until the bony anatomy is exposed. The decompression is now performed by the previous description in previous publications [11, 23]. The ipsilateral medial and inferior portion of the lamina is removed via a drill to facilitate access to the ligamentum flavum. It is recommended to start at the inferior medial edge of the lamina with the drill tip resting on the ligamentum flavum to sense the depth of the drill. The ipsilateral ligamentum flavum is removed from the insertion of the ligament cranially. Following a tilt of the operating table to improve the field of vision for the operating surgeon, an “over-the-top” laminotomy is performed to access the contralateral ligamentum flavum. After the removal of the hypertrophied ligamentum on the ipsilateral side, the thecal sac is identified. Importantly to note is the removal of the upper part of the caudal lamina to achieve adequate laminar decompression and avoid revision surgery. This is followed by repeating the above steps on the level above or below with a skin incision placed on the alternating side. This is repeated as many times as segments are involved in multisegmental LSS. Following irrigation and meticulous hemostasis with bipolar, the tubular retractor system is removed and the incision is closed (Table 1; Fig. 3).

**Table 1:** Ten-step outline of slalom unilateral laminotomy for bilateral decompression (sULBD) with navigation

Step #	Procedure
1	Patient positioning and localisation using a low-dose CT scan with calibration of navigation
2	Incision and placement of tubular retractor system using serial dilation
3	Removal of soft tissue, exposure of bony anatomy, and identification of inferior edge of the lamina
4	Curved drill with a 3 mm fluted matchstick drill bit and ipsilateral laminotomy
5	Identify the cranial insertion of the ligamentum flavum and removal of ipsilateral ligamentum flavum
6	Angle the tube contralaterally and rotation of table contralaterally.
7	“Over-the-top” laminotomy moving in contralateral direction. Removal of contralateral ligamentum flavum and removal of caudal part of lamina to prevent continued compression
8	Confirmation of adequate decompression of thecal sac in a clockwise fashion starting at 6 pm (Fig. 6) and using microscopic view and navigation
9	Repeat steps 2–8 on level above with an incision on the contralateral side
10	Hemostasis, irrigation (fibrilar/snow, epifix, gelfoam, steroids), removal of tubular retractor, and injection of muscle with local anesthetic

As with any type of spinal surgery, a potential complication includes leakage of CSF [11]. Briefly, if there is an opening big enough to allow a nerve root to protrude, we will perform primary closure with sutures: Scanlan dural repair set with a 4 − 0 Nurolon TF-5 “fishhook” needle, alternatively DuraStat and dural sealant. If the leak is very small, we use dural sealant materials and 24-hour bedrest following the intervention.

During the sULBD approach, we use navigation. The key steps for navigation include the planning of skin incisions, the confirmation of the correct placement of the correct tubular retractor, and confirmation of the exact localization of the ipsilateral pars which needs to be preserved throughout the decompression, especially higher up in the lumbar spine. Navigation has also been useful in confirming adequate decompression on the ipsilateral and contralateral sides. Navigation harbors the advantage of confirming and controlling the extension of decompression, above all for the contralateral side in our described over-the-top technique. On the contralateral side, the medial border of the caudal pedicle needs to be identified to confirm lateral recess decompression and identify the contralateral foramen. However, it is not possible to confirm the cranial pedicle on the contralateral or ipsilateral side (Fig. 3).

**Fig. 4:**
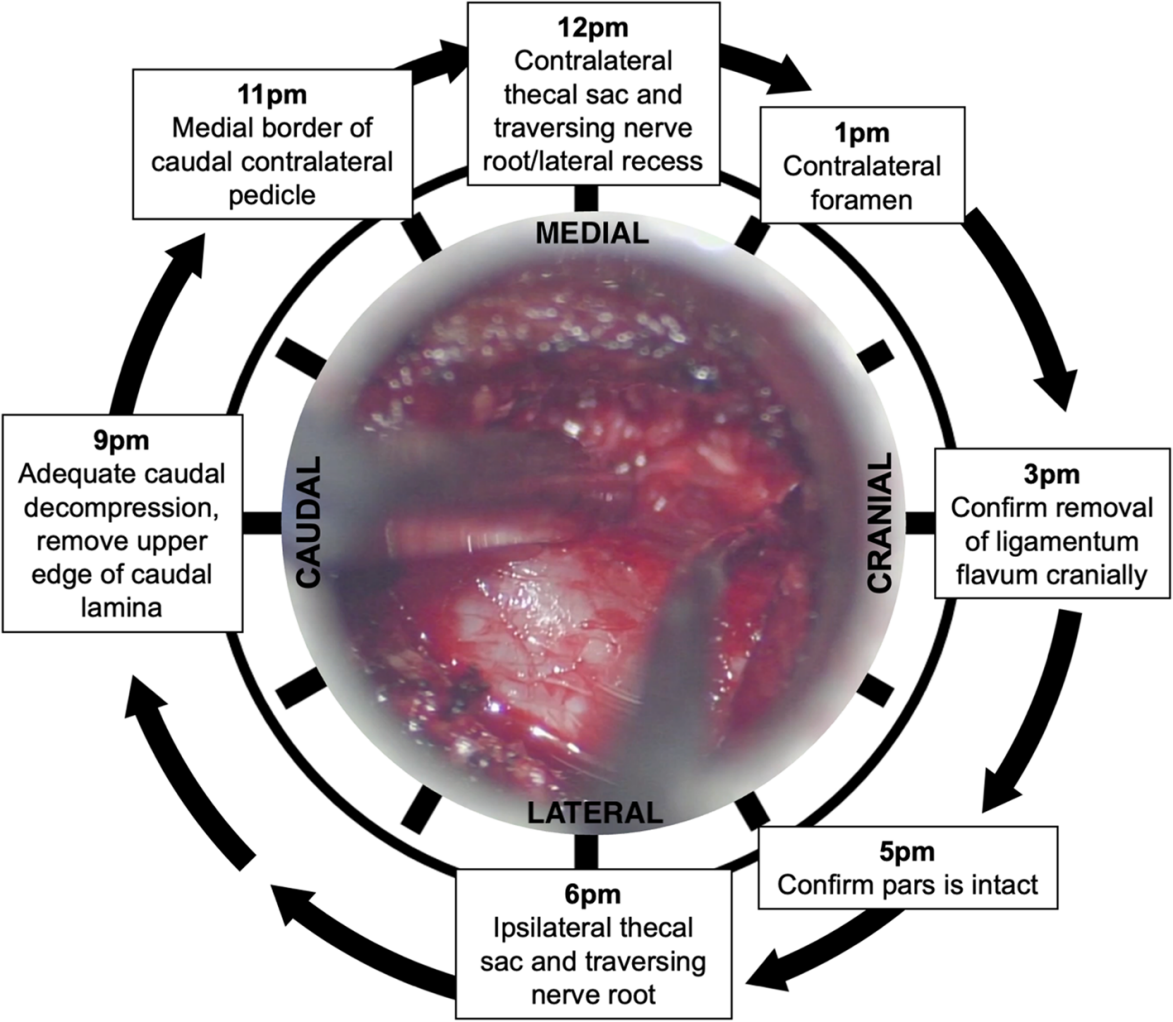
Confirmation of adequate decompression in clockwise technique from the right ipsilateral side before closure

Confirmation of adequate caudal decompression is crucial: The upper few millimeters of the caudal lamina should always be removed as there can be significant buckling of the ligamentum flavum that may cause thecal sac compression. We recommend confirmation of adequate decompression in clockwise direction (Fig. 4). The level of decompression should be determined on saggital imaging. Previous work from our group has shown that in patients with bilateral clinical symptoms from LSS, a unilateral approach for bilateral decompression will result in adequate decompression and symptom resolution on both sides [24].

### Navigation

The sULBD was performed with navigation due to its multifold advantages: For the surgical team members, the radiation exposure is eliminated and during the procedure, no lead has to be worn further reducing strain on the team members. After the imaging has been obtained the surgeon can navigate each level and every step of the procedure without the need for an x-ray tech or bringing fluoroscopy in and out. The images obtained via an initial low-dose CT scan can be transferred to a navigation software and enable to surgeon to retrieve anatomical information beyond the confirmation of the spinal level. With modern intraoperative CT technology, patient radiation can be minimized. 3D information includes confirmation of the correct side of tube placement. The pars interarticularis can be identified and protected and adequate decompression can be confirmed cranially and caudally by placing a navigation pointer intraoperatively. Placement of the reference array usually is 3 min, the time of a spin is 5 min, so the total time would amount to a maximum of 10 min. However, it eliminates the time required to bring in fluoroscopy several times for each level treated, so overall navigation saves time for multilevel cases and facilitates the workflow. Overall, using navigation facilitates the workflow by reducing the number of times fluoroscopy needs to be brought in – this becomes particularly relevant with the number of spinal levels involved in treating multi-segmental LSS. Even for the skilled and experienced surgeon, navigation harbors important advantages including the elimination of radiation exposure to the surgical team, further visualization of key anatomical landmarks during the procedure, and a simplified workflow intraoperatively (Table 2).

**Table 2:** Benefits of navigation in the sULBD approach

#	Description
1	Eliminates radiation for the surgical team. Radiation for patient can be minimized with modern intraoperative CT systems.
2	Surgical team does not need to wear lead thus eliminating the burden of the weight of lead
3	Beyond confirmation of spinal level, navigation allows the visualization of anatomical structures and all relevant landmarks for the ULBD
4	Enables identification and protection of the pars interarticularis, especially in upper lumbar spine (Fig. 3)
5	Simplify the workflow by reducing the number of fluoroscopy/x-ray technicians, esp. in multi-segmental LSS
6	Confirmation of adequate decompression on contralateral side, and cranially/caudally of involved segment
7	Facilitates the learning curve of the sULBD approach

### Data collection

We conducted the data collection phase during the study period from November 1st to November 30th, 2022. Demographic variables collected include age, sex, and BMI. Clinical variables collected include presenting symptoms, operative time defined as time from incision to wound closed, spinal levels operated on, EBL, LOS, complications, and follow-up period. We also collected per segment blood loss and operative time. The primary outcomes of this study were a reduction in VAS for back pain and leg pain and ODI. We report secondary outcomes including i) symptoms on clinical presentation, ii) operative time, iii) EBL, iv) LOS, v) and follow-up.

### Data analysis

Data analysis was conducted in Microsoft Excel (Version 2202 Build 16.0.14931.20806). We report categorical variables as absolute numbers and percentages and continuous variables as mean and standard deviation. Due to the low number of patients eligible to be included in our study, we adjusted our data analysis for the primary outcomes based on the standard error of the mean [25]. Therefore, we report VAS and ODI as the mean and the standard error of the mean.

## Results

From January 2020 until February 2022, seven patients (four females and three males) underwent a sULBD at our institution. The average age was 70.86 years and the average BMI was 26.43 kg/m². Four patients presented with isolated back pain, one with back pain and weakness, one with weakness, one with hip/buttock and leg pain, and one patient experienced incontinence. The most common spinal levels operated on in this cohort were L3-L5 (4 patients). Less commonly involved were the spinal levels L2-L4, L1-S1, and L4-S1 (one patient each). The average operative time was 196.14 ± 54.45 min. The mean operative time per segment was 94.71 ± 31.53 min. The mean estimated blood loss (EBL) was 105.83 ± 98.76 mL. The mean EBL/segment was 45.12 ± 49.52 mL. The average hospital length of stay (LOS) was 1.67 ± 1.11 days and no major surgical complications occurred (Table 3).

**Table 3:** Patient demographics and outcomes

	*N* = 7
Female	4 (57.1) ^a^
Age (in years)	70.86 ± 5.67 ^b^
BMI (kg/m²)	26.43 ± 3.53 ^b^
**Clinical presentation**	
Back pain	5 (71.4) ^a^
Weakness	2 (28.6) ^a^
Incontinence	1 (14.3) ^a^
**Operative variables and follow-up**	
L1-S1, five levels	1 (14.3) ^a^
L2-L4, two levels	1 (14.3) ^a^
L3-L5, two levels	4 (57.1) ^a^
L4-S1, two levels	1 (14.3) ^a^
Operative time (in minutes)	196.14 ± 54.45 ^b^
Operative time per segment (in minutes)	94.71 ± 31.53 ^b^
Estimated blood loss (in mL)	105.83 ± 98.76 ^b^
Estimated blood loss per segment (in mL)	45.12 ± 49.52 ^b^
Length of stay (in days)	1.67 ± 1.11 ^b^
Follow-up (in months)	20.71 ± 9.00 ^b^
**Outcome measures**	
Back pain VAS, pre-OP	4.71 ± 1.17 ^c^
Back pain VAS, post-OP (short-term)	1.58 ± 0.69 ^c^
Back pain VAS, post-OP (long-term)	1.50 ± 0.8 ^c^
Leg pain VAS, pre-OP	4.33 ± 1.37 ^c^
Leg pain VAS, post-OP (short-term)	1.70 ± 0.77 ^c^
Leg pain VAS, post-OP (long-term)	1.21 ± 0.91 ^c^
ODI, pre-OP	33% ± 7 ^c^
ODI, post-OP (short-term)	21% ± 7 ^c^
ODI, post-OP (long-term)	12% ± 6 ^c^

*Abbreviations:* *ODI* Oswestry Disability Index, *VAS* Visual analogue scale

^a^reported as absolute (relative)

^b^reported as mean ± SD

^c^reported as mean ± standard error of the mean

**Fig. 5:**
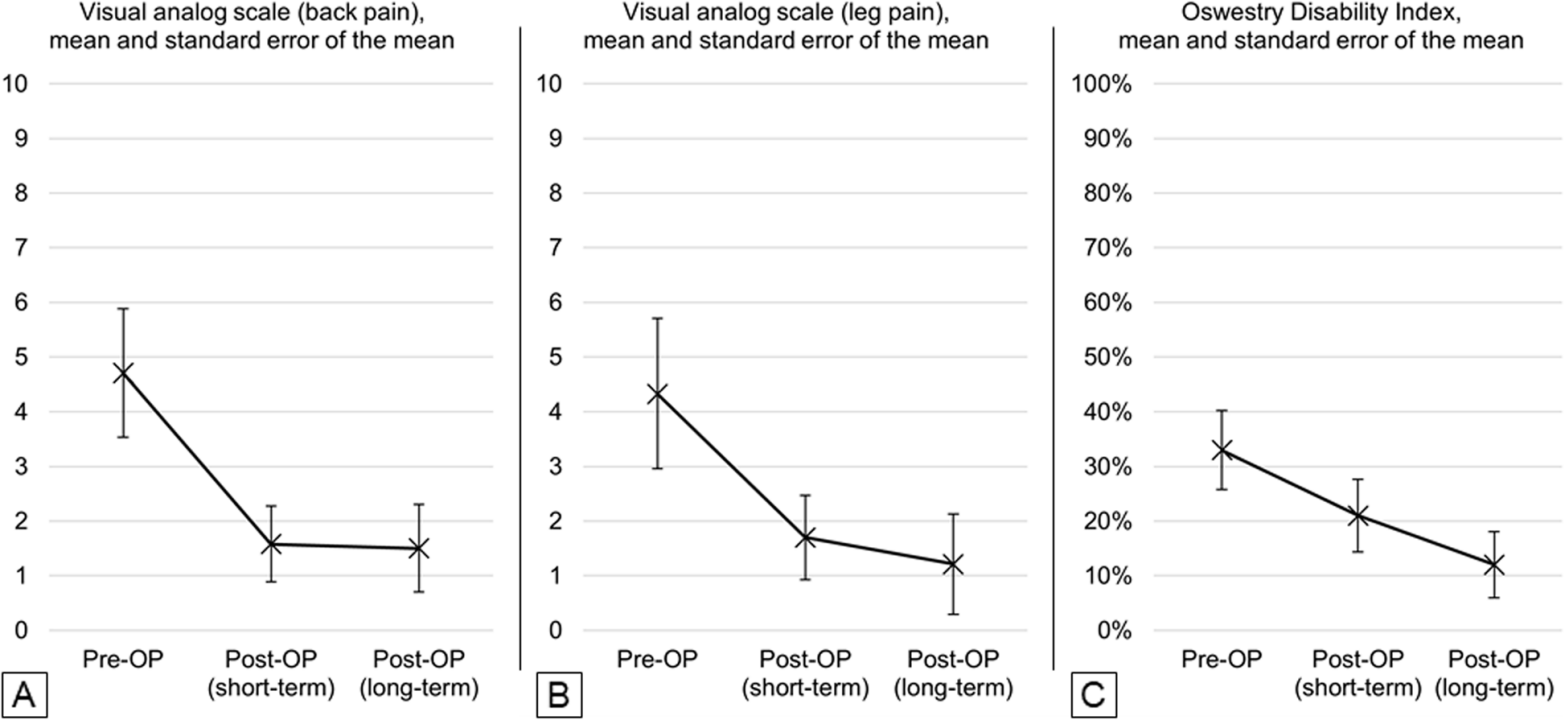
Primary outcomes – VAS and ODI – preoperatively, within short-term (mean: 0.68 months), and long-term (19.05 months) follow-up. Abbreviations: ODI = Oswestry Disability Index, VAS = Visual analogue scale

Patients were followed for an average of 20.71 months. Using the visual analog scale (VAS), back pain was reduced from 4.71 ± 1.17 (standard error of the mean) to 1.58 ± 0.69 on short-term follow-up of 0.68 months on average and 1.50 ± 0.80 on long-term follow-up of 19.05 months on average (Fig. 5A). Leg pain was scored as VAS of 4.33 ± 1.37 pre-operatively and was reduced to a VAS of 1.70 ± 0.77 on short-term follow-up and 1.21 ± 0.91 on long-term follow-up (Fig. 5B). The mean pre-operative Oswestry Disability Index (ODI) score was 33% ± 7. Mean postoperative ODI scores were 21% ± 7 on short-term follow-up. Mean postoperative ODI scores were 12% ± 6 on long-term follow-up (Fig. 5C).

## Discussion

Various pathologies of the spine can cause low back pain with radiating pain. Such pathologies causing radiculopathy include LSS, disc herniations, and spondylolisthesis [26]. To relieve pressure on the spinal cord and nerves in clinically worsening conditions or emergency settings, adequate decompression of affected segments has been established as the standard of care. To achieve a less invasive approach, yet maintaining high accuracy, we performed a slalom unilateral laminotomy for bilateral decompression with navigation. Herein, we outline the fundamental ten steps to accomplish the sULBD – a MIS surgical technique that is safe and effective in achieving decompression of multisegmental LSS [18, 19].

Minimally invasive techniques have been associated with a complex learning curve. Strategies to accelerate the learning curve include the understanding of three-dimensional anatomy, surgical aptitude along with manual dexterity, and guidance by a proficient surgeon along the way [27, 28]. Minimally invasive techniques including the tubular approach for decompression are complex and as with other complex surgical procedures require a learning curve. Navigation can aid in speeding up this process through adding a layer of safety to the surgeon as navigation can confirm and control the extent of decompression. It has been suggested that after twenty to thirty procedures performed by a single surgeon, the operative time and results reach a more consistent stage on the learning curve [29]. Despite a higher complication rate at the beginning of the learning curve, clinical and radiographic long-term outcomes are not affected by the learning curve [30]. The use of virtual and augmented reality and surgical simulators are strategies to flatten the learning curve [31]. Recently we published our experience with surgical simulation for ULBD – Melcher et al. described the deviation of the ULBD approach in terms of attention to technical skills, skipped steps, the occurrence of errors, and timing. Using a simulation model for training purposes, procedural time could be reduced, and surgical errors could be reduced significantly [17].

To contribute to minimizing the learning curve, our group has demonstrated various surgical techniques in the field of MISS [11, 22, 32–34]. Using ten-step guides, we have broken down several novel procedures into easy-to-adapt procedures. Using ten steps, we have demonstrated the MIS surgical technique to achieve microsurgical tubular laminotomy, microsurgical foraminotomy, microsurgical tubular discectomy, and microsurgical resection of lumbar synovial cysts [11]. In patients with central disc herniations, a generous over the top decompression needs to be completed first in order to create room for the subsequent steps. The ULBD approach for a giant disc herniation has previously been described in a ten-step approach [35].

### ULBD approach

The concept of using a unilateral approach to achieve bilateral decompression has been introduced in the 1990s [6, 36, 37]. Following patient positioning, localization of the spinal level, and placement of the reference array and navigation (Steps 1 and 2), a tubular retractor is placed (Step 3), and soft tissue is removed (Step 4). Ipsilateral laminotomy and subsequent resection of the ligamentum flavum is performed (Step 5 and 6). Contralateral decompression is achieved via “over-the-top” drilling (Step 7). After the final inspection, hemostasis is initiated (Steps 8 and 9). The tubular retractor is slowly removed and the fascia is reapproximated before being closed (Step 10) [32].

Cost estimates for the time in the operating room are around 66 USD/minute. Decreasing the operative time is therefore not only in the interest of the patient but also an achievement towards health economics [38].

In a twelve-step guide, the lumbar endoscopic ULBD (LE-ULBD) has been shown to be safe and effective [39]. Endoscopic ULBD approaches have been hypothesized to reduce the invasiveness of the procedure even further: Hua et al. reported a shorter operative time, estimated blood loss, and length of hospital stay in a cohort of endoscopic ULBD compared with a cohort treated with the MIS-TLIF technique. Clinical outcomes in both groups comprising a total of 112 consecutive patients were rated as excellent/good according to the modified Macnab criteria in > 90% of patients and no significant differences in clinical outcomes were reported [40].

### Previous clinical outcomes in the ULBD approach

The surgical time reported in our case series is comparable with previous studies: Arai et al. prospectively analyzed 50 patients undergoing the ULBD approach [10]. They report a mean surgical time of 181 min where as we report a mean surgical time of 196 min. The mean estimated blood loss was 114 mL whereas we report a mean EBL of 106 mL. Importantly to note however, 25/50 patients underwent a single-level ULBD whereas we report solely multilevel spinal decompressions using the slalom ULBD technique reported herein.

Intraoperative navigation offers advantages over fluoroscopic techniques which require frequently bringing the C-arm into the surgical field. Firstly, navigated surgeries reduce intraoperative radiation exposure compared with fluoroscopically assisted surgeries [21]. Secondly, better accuracy is achieved by using intraoperative navigation systems. Despite a perceived higher intraoperative time when using navigation [41], a randomized clinical trial showed shorter operating times when compared with conventional surgery [42].

### Decision-making rationale behind choosing a side for the approach

**Fig. 6:**
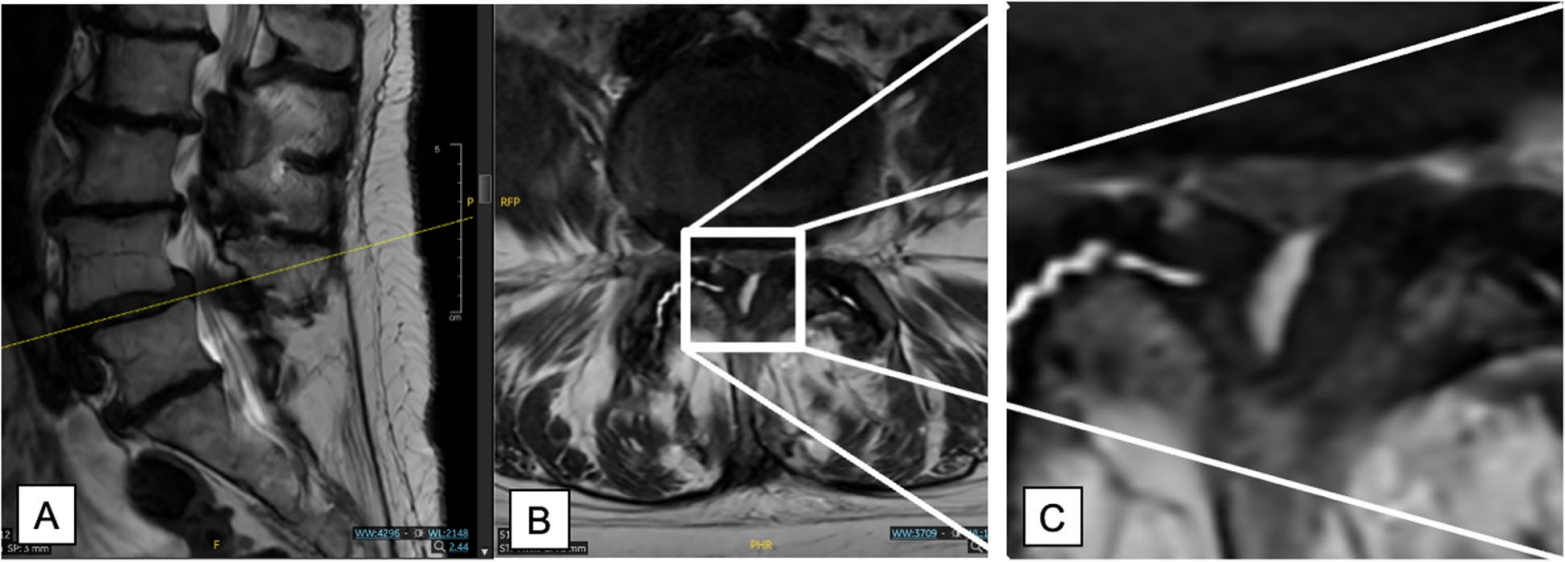
Decision-making on which side to use the unilateral approach based on a case example of calcified ligamentum flavum (LF). In this case, it is recommended to use the approach via calcified right side as the ligamentous structures are more angled on the right side as compared to the smooth left side; a dural tear is more likely to occur on the right side allowing for a better field of view. It is easier to fix a CSF leak from the ipsilateral side. Intraoperatively, calcification of the dura was confirmed

Depending on the variable anatomy of both sides, the approach should be chosen carefully. It may be advantageous to approach a particular anatomy from a specific side. In patients with mild scoliosis, the approach from the convex side is recommended to prevent further collapse of the concave side. In patients requiring a foraminotomy, we will approach that level from the contralateral side. In patients with a synovial cyst, we currently prefer an ipsilateral approach since a potential CSF leak can be closed more effectively from that side. In patients with calcifications of the ligamentum flavum, we will also choose an ipsilateral approach because there is a higher risk of encountering a CSF leak that may require primary closure. These calcifications should be suspected in patients who have asymmetric ligamentum flavum thickening or where the ligamentum flavum thickening on one side is not smooth (Table 4). This may be visualized on preoperative or intraoperative imaging. In a case operated at our institution, calcification of the ligamentum flavum was identified on preoperative imaging (Fig. 6) and could be confirmed intraoperatively.

**Table 4:** Approach Pearls: How to choose the side for the approach?

Patient population	Side and surgical rationale
Mild scoliosis	Approach from convex side, to prevent further collapse of concave side
Central stenosis with neurogenic claudication	Right-sided approach for right-handed surgeon and left-sided approach for left-handed surgeon –1–2 levels: one incision –3–4 levels: sULBD
Unilateral disc herniation	Ipsilateral approach
Additional foraminotomy required in foraminal stenosis with radiculopathy	Contralateral approach
Synovial cyst	Ipsilateral approach, to manage a potential CSF leak effectively
Calcified ligamentum flavum (Fig. 5)	Ipsilateral approach, to manage a potential CSF leak effectively
Symptomatic lateral recess stenosis	L4/5/S1: Ipsilateral Approach; L1/2/3/4: Contralateral approach to avoid ipsilateral pars injury

### Surgical pitfalls in the decompression of the contralateral side

Adequate visualization of the contralateral side can be achieved by tilting the operating table away from the surgeon and placing the tubular retractor medially. Then, the spinous process and lamina are undercut on the contralateral side using the drill. The ligamentum flavum is initially spared to protect the dura. Once the midline is identified by the presence of epidural fat, a nerve hook is inserted to demonstrate the two leaves of the ligamentum flavum which is then removed by Kerrison rongeurs. Upon complete removal, dural exposure and decompression are achieved on the contralateral side (Fig. 3F) [43].

If a unilateral incision is indicated, the decision-making on which side to choose requires a careful review of preoperative imaging (Table 4). If a calcified ligamentum flavum is seen, it is recommended to use the approach via this side. In one case operated at our institution, we identified a calcified ligamentum flavum on the axial view which appeared more angled on the right side as compared with the smooth, uncalcified left side (Fig. 6). Since a dural tear is more likely to occur on the right side due to the porous substance of the calcified ligament, using the approach on the right side allows for a better field of view in case dural repair becomes necessary. Intraoperatively, calcification of the dura was confirmed.

### Limitations

Finally, several limitations arise from the methodology of our study. Since the sULBD technique involves the decompression of multiple spinal levels, the number of potential patients is lower in the first place. The number of patients in our cohort was low despite of the long study period. First, we defined narrow inclusion criteria to reduce the heterogeneity of our cohort while including enough patients to report the safety and efficacy of our presented technique. Second, we determined a minimum follow-up of nine months to ensure that patients achieved long-term relief from the surgery. One patient was diagnosed with multiple sclerosis multiple years before his sULBD surgery and ODI scoring in this patient might have been affected by relapse phases. Short-term outcomes should therefore be treated with careful consideration of the clinical context. Nevertheless, we included outcomes as part of this study despite our main intention to report the safety and efficacy of the technique described herein. Intraoperative CT imaging generates less radiation to the surgical team but more to the patient. We addressed this limitation by using modern AIRO CT which minimizes the amount of radiation.

Nonetheless, our report focused on describing the ten steps needed to achieve a safe and effective decompression of multi-segmental LSS using the sULBD approach. We are confident that the technique described herein might encourage future studies to report larger patient samples. In order to minimize radiation to the patient, we are currently working on technology to merge preoperative MRI scans with intraoperative antero-posterior and lateral fluoroscopy cuts.

## Conclusion

Here, we outline the fundamental ten steps in achieving a slalom unilateral laminotomy for bilateral decompression (sULBD) with navigation for the treatment of multisegmental lumbar spinal stenosis (LSS). The sULBD procedure involves ten stages, including CT scan and navigation setup, incision and tubular retractor setup, soft tissue removal and inferior lamina edge identification, ipsilateral laminotomy using a drill, ligamentum flavum removal, contralateral laminotomy, removal of ligamentum flavum extensions, confirming thecal sac decompression using navigation and microscopy (Fig. 4), repeating on the upper level via contralateral incision, and concluding with hemostasis and closure. Importantly, navigation enhances safety and efficiency. Compared to the standard sULBD technique, the incorporation of navigation provides anatomic localization without exposure to radiation for a higher safety profile along with a fast and efficient workflow.  

## Data Availability

The datasets used during the current study available from the corresponding author on reasonable request.

## Abbreviations

EBL: Expected blood loss
LF: Ligementum flavum
LOS: Length of stay
LSS: Lumbar spinal stenosis
MISS: Minimally invasive spinal surgery
ODI: Oswestry Disability Index
sULBD: Slalom unilateral laminotomy for bilateral decompression
ULBD: Unilateral laminotomy for bilateral decompression
VAS: Visual Analogue Scale
